# Repetitive elements in parasitic protozoa

**DOI:** 10.1186/1741-7007-8-64

**Published:** 2010-05-24

**Authors:** Christine Clayton

**Affiliations:** 1 Zentrum für Molekulare Biologie der Universität Heidelberg, DKFZ-ZMBH Alliance, Im Neuenheimer Feld 282, D-69120 Heidelberg, Germany

## Abstract

A recent paper published in *BMC Genomics *suggests that retrotransposition may be active in the human gut parasite *Entamoeba histolytica*. This adds to our knowledge of the various types of repetitive elements in parasitic protists and the potential influence of such elements on pathogenicity.

See research article http://www.biomedcentral.com/1471-2164/11/321

## Commentary

The genomes of eukaryotes contain numerous types of repetitive element with a wide variety of functions. Some are within coding regions; others are in untranslated regions of mRNAs or are located in regions that are important for chromosome maintenance; and many may have no function at all beyond self-perpetuation. Repetitive elements fall into three broad classes. Simple repeats can change in copy number, but do not move to new locations; DNA 'cut-and-paste' transposons are able to 'jump' to a new location; and retroposons move via an RNA intermediate, leaving an intact retroposon at the original location. Changes in the numbers, or locations, of repetitive elements can alter the structures of proteins, influence gene expression and affect chromosome segregation and karyotypes. Repetitive elements are therefore significant drivers of diversity.

There is currently little direct evidence for active transposition of the elements present in the genomes of parasitic protists but a genome-wide bioinformatic screen of the sequenced genome of the gut parasite *Entamoeba histolytica *published recently in *BMC Genomics *by Huntley *et al*. [1] has found indirect evidence of recent transposition events involving the SINE (short interspersed nuclear element) class of retroposon.

The numbers of simple repeats and transposons in the genomes of parasitic protists vary considerably, with estimates of the proportion of repetitive DNA in genomes varying from 11% to 65%. These numbers are unreliable, however, as repetitive regions usually become compressed during alignment, and sequences present in multiple locations cause difficulties in assembly of contiguous chromosomes. Within species, variations between isolates in the numbers of simple repeats, and in the locations of transposable elements, are useful for epidemiological studies. Multicopy sequences are also ideal targets for amplification-based diagnostics.

## Simple repeats

**Figure 1:**
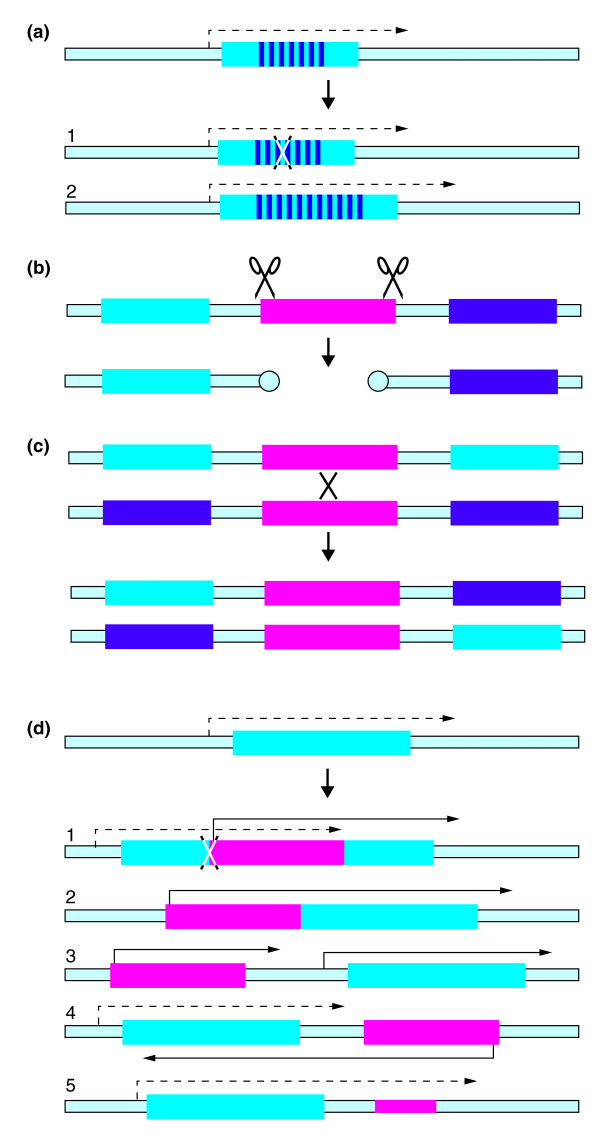
**Effects of repeated sequences on gene expression**. **(a) **An open reading frame (cyan rectangle) with a repetitive region (dark-blue stripes) is transcribed from an upstream promoter (dotted line and arrowhead). 1, Replication slippage leads to a frameshift with a new stop codon (indicated by the cross over the open reading frame). 2, Replication slippage or unequal recombination can increase the number of repeats. **(b) **A mobile element (magenta gradient shading) is cut from between two genes (cyan and purple rectangles) by transposase (scissors). An error in repair after transposition introduces a chromosome break, which is repaired to give new telomeres (circles). **(c) **Recombination between homologous mobile elements on two different chromosomes leads to a translocation. **(d) **Effects of a mobile element (here shown with its own promoter and transcription) on gene expression. 1, An insertion within an open reading frame results in a truncated protein. 2, An insertion at the beginning of an open reading frame can result in large amounts of a fusion protein. 3, The presence of the retroposon or transposon promoter can activate the transcription of a downstream gene, for example by opening the chromatin. 4, Insertion in an opposite orientation results in antisense RNA. 5, Insertion in a 3'-untranslated region can affect mRNA processing, stability or translation. The example shown has normal monocistronic transcription; for kinetoplastids, transcription is polycistronic (not shown).

Simple repeats are classified into 'microsatellites' - repeats of 1 to 6 nucleotides - and longer repeats. Within eukaryotic open reading frames (ORFs), they are found in genes encoding fibrous and cytoskeletal proteins. Protist parasite surface antigen genes can also be repetitive: examples include a trypanosome surface protein consisting largely of Glu-Pro repeats, and the circumsporozoite proteins of *Plasmodium*. Repeats in intergenic regions can affect chromatin structure or chromosome segregation; for example, repeats are sometimes present at centromeres. Simple repeats expand, contract and mutate through recombination and replication slippage. Within ORFs, errors may result in frame-shifting and premature stop codons (Figure 1a, 1); those that conserve the ORF change the length of the protein (Figure 1a, 2), often (but not always) without major functional consequences.

## DNA transposons

Eukaryotic DNA transposons have terminal inverted repeats, between which lies an ORF that encodes the transposase, the protein required for transposition. The endonuclease activities of transposases both recognize the inverted repeats at the ends of the transposon and cut the DNA target site in a staggered fashion, leaving single-stranded ends. The transposed segment is inserted at the cut, and ligated. Filling-in of the single-stranded segments creates short genome duplications at the insertion site. This type of cut-and-paste transposition moves the transposon but does not increase the copy number; mechanisms involving replication of the transposon during transposition also exist among DNA transposons. Damage to the inverted repeats immobilizes the transposon, but elements with intact terminal repeats can move if the transposase is provided in *trans*.

*E. histolytica *is a digestive tract parasite that causes severe diarrhoea and lethal abscesses. The sequenced isolate of *E. histolytica *has Mutator and mariner-like DNA transposons, and about 800 fragmented copies of a novel element called EhERE1; this has 2.2-kb inverted repeats surrounding a 2.7-kb ORF that encodes a protein with weak similarity to ATPases involved in chromosome segregation and DNA repair [2]. An additional element, EhERE2, with an ORF of unknown function, is unique to *E. histolytica*. Other *Entamoeba *species have ERE1 and other types of DNA transposons [2,3].

## Retroposons

The second large class of transposable elements in eukaryotes are the retroposons (retrotransposons), which move within genomes via an RNA intermediate. The so-called LTR retroposons terminate in long terminal repeats (LTRs) and are similar in structure to genomically integrated retroviruses. LTR retroposons are absent in many parasitic protist genomes, whereas non-LTR retroposons (which do not have the LTRs) are widespread.

An intact (or autonomous) non-LTR retroposon encodes an endonuclease and reverse transcriptase, which are required, respectively, for nicking the target DNA site and for copying the retroposon RNA into DNA during retroposon insertion. As with DNA transposons, transposition results in a duplication of the target DNA at the insertion site. Retroposons that lack an intact ORF, but have intact ends, can move using relevant enzymes encoded elsewhere in the genome. Insertion sites may be nonspecific or show very weak conservation, such as being enriched in particular nucleotides [1]. Retroposons and genes encoding reverse transcriptase and/or endonuclease are found in apicomplexans [4], *Trichomonas *[5], kinetoplastids [6], *Entamoeba *[2] and *Giardia *[7].

Two ubiquitous classes of eukaryote non-LTR retroposons are the LINEs (long interspersed nuclear elements) and the SINEs. LINEs are typical non-LTR retroposons: the genomic element is transcribed into RNA by RNA polymerase II from a promoter at the 5' end of the LINE. The RNA encodes reverse transcriptase and endonuclease, which mediate transposition. SINEs are much shorter than LINEs as they lack the ORF; they originate from RNA polymerase III transcripts and rely on LINE-encoded enzymes for reverse transcription and genomic integration.

*Entamoeba *species have several types of LINEs, which probably diverged from a common ancestor; more than 740 copies constitute 11% of the *E. histolytica *genome [2]. The 88 complete LINEs have two ORFs: they encode one protein of unknown function, and one with reverse transcriptase, nucleic-acid binding and endonuclease domains. No *E. histolytica *LINE has both ORFs intact [2], but - assuming that both are required for retrotransposition - proteins encoded by different elements may function together. There are 750 copies of three related SINEs, of which about 370 have intact ends. The LINEs and SINEs tend to be clustered together in the genome, sometimes with DNA transposons as well [2]. In their recent study, Huntley *et al*. [1] developed a hidden Markov model for *Entamoeba *SINE-like elements in order to be able to annotate both intact and truncated copies reliably. They used the model to scan the *E. histolytica *genome for SINE-like elements, then classified them according to repeat structure, and the boundary sequences, in order to be able to detect evidence suggestive of recent transposition. They found 393 SINE1 elements. SINE1s vary in length owing to the presence of variable numbers of repeats of 26 to 27 bp, but the 5'- and 3'-terminal regions are conserved; transcripts are abundant and possible polymerase III promoter elements can be identified [1].

Other parasites also have retroposons: for example, *Giardia *has three, of which two encode reverse transcriptase [7]. They are found in various locations, including telomeres, where copy-number variation is partially responsible for the differences in size between chromosomal homologues [7,8]. Trypanosomes and leishmanias have hundreds of copies of a long autonomous LINE-like retroposon called 'ingi' [6] that encodes a multi-function reverse transcriptase/endonuclease/RNaseH. There are also multiple truncated and mutated forms. Ingi elements are scattered in clusters across the chromosomes [6], sometimes marking centromeres. A conserved ingi-specific 77-bp terminal sequence was shown to function as an RNA polymerase II promoter in *Trypanosoma cruzi *[9].

The presence of transposons and retroposons in multiple copies in opposite orientations can result in the generation of double-stranded RNA (dsRNA). In many eukaryotes, including African trypanosomes [10] and *Giardia *[8], the cellular RNA interference (RNAi) machinery processes the dsRNA to make short-interfering RNAs, which target retroposon transcripts for degradation through RNAi. In RNAi-deficient trypanosomes, the levels of retroposon-derived RNAs were considerably increased, and new copies of a retroposon were seen in the genome [10]. This is currently the only direct evidence for transposon movement in parasitic protists.

The presence of identical retroposons in different places can, however, be interpreted as indirect evidence for recent transposition, especially if the target-site duplications are intact, as there is no known selective pressure for retention of the target site duplication. Huntley *et al*. [1] found 15 SINE1s with intact target-site duplications and, following the above logic, suggest that these SINE1s are recent transpositions. It is therefore possible that retrotransposition is still active in *Entamoeba*.

## The influence of repetitive elements on gene expression

The movement and amplification of transposons affects genome structure. Amplification increases the amount of DNA that has to be replicated in every cell cycle, whereas errors in nick repair can cause chromosome breaks (Figure 1b). The presence of multiple copies of a similar transposon at different locations facilitates homologous recombination between chromosomes, resulting in translocations (Figure 1c). Similarly, in African trypanosomes, patches of conserved simple repeats near the telomeres provide sites for recombination during antigenic variation [11]. Recombination between repeats in *cis *on the same chromosome causes duplications and internal deletions.

Transposons can also influence gene expression. Chromosome rearrangements alone can have epigenetic effects on transcription of nearby genes. The insertion of a transposon or retroposon within an ORF can truncate it (Figure 1d, 1) or result in production of large amounts of a fusion protein (Figure 1d, 2), which might either retain activity or have a dominant-negative effect. Transposon promoters can activate transcription of downstream genes (Figure 1d, 3), either by opening chromatin, or by readthrough if (as in kinetoplastids) transcription is polycistronic. Transposon promoters can also result in production of antisense RNA (Figure 1d, 4). The immobile ingi-related 'SIDER' retroposons of *Leishmania *have, however, been 'domesticated' to regulate mRNA levels at the post-transcriptional level. SIDER is found in 3'-untranslated regions of many mRNAs (Figure 1d, 5), where its presence correlates with low mRNA abundance and translational repression [12]. Insertions in untranslated regions or introns could also influence mRNA splicing or polyadenylation. Any of these changes could change the levels of pathogenicity factors or influence parasite growth and differentiation.

Evidence so far suggests that the pathogenicity of *E. histolytica *isolates varies extensively. The study by Huntley *et al*. [1] shows that that movement of retroposons might contribute to this variability.
